# Supplementary material for: Netcombin: An algorithm for constructing optimal phylogenetic network from rooted triplets
Source: PLoS One. 2020 Sep 18;15(9):e0227842. doi: 10.1371/journal.pone.0227842 (PMC7500971; doi:10.1371/journal.pone.0227842)
Supplement: S4 File — (ZIP) [file pone.0227842.s004.zip › SequenceData-Triplet/sequenceData-10-20-30-0-Triplet/length 300, numseq 40, rate 10^-7/1 seq.pdf]

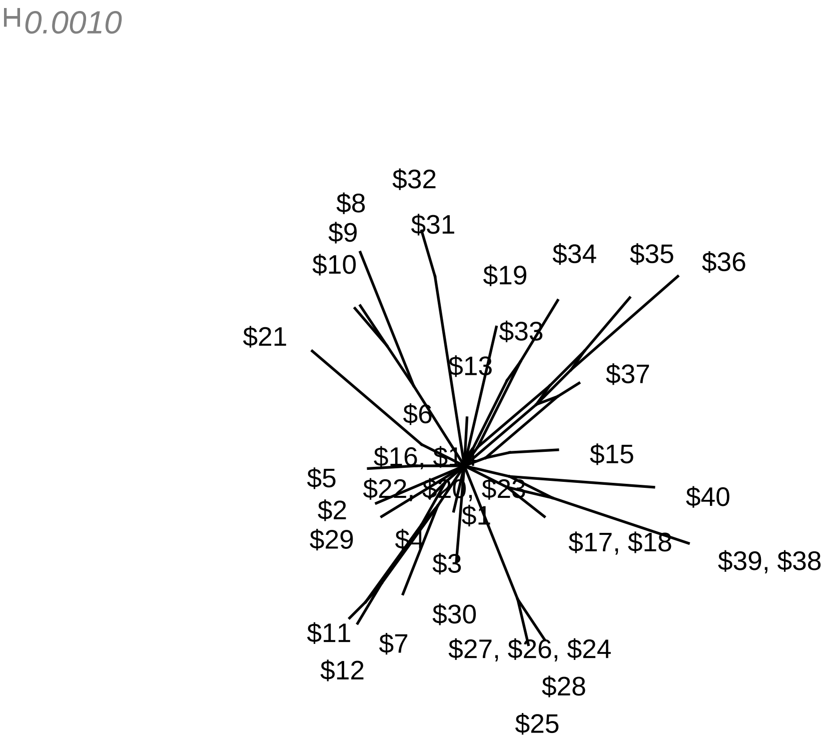
0.0010
$32
$8
$31
$9
$10
$34
$35
$36
$19
$21
$33
$13
$37
$6
$15
$16
$5
$22, $20, $23
$40
$2
$1
$29
$4
$17, $18
$3
$39, $38
$30
$11
$7
$27, $26, $24
$12
$28
$25
